# Challenges in the Diagnosis of Systemic Lupus Erythematosus in an Adolescent With Sexually Transmitted Infections: A Case Report and Review of the Literature

**DOI:** 10.7759/cureus.101611

**Published:** 2026-01-15

**Authors:** Alison Brittain, Monica I Ardura, Megan Brundrett, Vidya Sivaraman

**Affiliations:** 1 Pediatric Rheumatology, Nationwide Children's Hospital, Columbus, USA; 2 Pediatrics/Infectious Disease, Nationwide Children's Hospital, Columbus, USA; 3 Primary Care, Nationwide Children's Hospital, Columbus, USA

**Keywords:** autoimmunity and infection, hiv, sexually transmitted infections, syphilis, systemic lupus erythematosus

## Abstract

Systemic lupus erythematosus (SLE) is often referred to as the great imitator, as it can mimic the presentation of several diseases, including infections. We present the case of an 18-year-old male adolescent with clinical and laboratory findings of SLE who was later found to have concurrent syphilis, gonorrhea, chlamydia, and human immunodeficiency virus (HIV) infections. Diagnosis of these concurrent infections was complicated by symptom overlap between these diseases, primarily between SLE, HIV, and syphilis, as well as positive autoantibody tests. Treatment for lupus was stopped after infections were found; however, thorough retesting for SLE has not been performed, as the patient was lost to follow-up. This case highlights the need to keep a broad differential when diagnosing and treating patients with SLE, as false-positive serologic tests for SLE may be present in individuals with infections. This case also underscores the need to perform screening for sexually transmitted infections (STIs) in individuals who are being considered for a rheumatologic diagnosis, especially before starting immunosuppressive therapies.

## Introduction

The diagnosis of systemic lupus erythematosus (SLE) is made based on clinical symptoms, pathologic features, and laboratory findings. The most recent guidelines from the joint American College of Rheumatology (ACR) and European League Against Rheumatism (EULAR) have improved the sensitivity and specificity of diagnosing SLE [1]. Despite these updated criteria, the presenting symptoms of SLE are non-specific and may be mistaken for or overlap with other etiologies, including infections or malignancies. 

Previous literature has highlighted the difficulty in diagnosing SLE when comorbid infections are present, particularly due to the strong symptom overlap between SLE and infections [2]. SLE can present with fever, malaise, lymphadenopathy, arthralgia, and oral ulcers, symptoms that are often present in infections, leading to uncertainty either at the time of diagnosis or during subsequent flares. Patients with SLE also have an increased rate of infections, suspected to be due to either immune dysregulation or immunosuppressive medications [3]. Infections can also cause transient positivity in autoantibody testing, a well-documented phenomenon particularly in human immunodeficiency virus (HIV) infections [4]. A significant amount of effort has been dedicated to developing clinical tools that help differentiate between lupus flares and infections [5,6]. Despite these advances, diagnosing a patient with SLE or a flare of SLE remains a challenge for rheumatologists. 

Here, we present the case of a male adolescent whose diagnosis of SLE was complicated by multiple concurrent sexually transmitted infections (STIs). This case highlights the need to be vigilant for the presence of infections during the initial diagnosis of SLE. After a diagnosis is made, the managing physician must remain mindful that co-occurring infections may masquerade as symptoms of an SLE flare and that re-screening for these infections may be necessary depending upon risk factors. 

## Case presentation

An 18-year-old African American male adolescent presented to his pediatrician with a chief complaint of migratory arthralgias that were worse in the morning, as well as chronic intermittent abdominal pain for two years, decreased appetite, and 10-pound weight loss. He had previously tested negative for celiac disease and *Giardia* and *Helicobacter pylori* infections. He reported being sexually active with both male and female partners with consistent condom use. He reported having been tested for STIs two weeks prior with negative tests for syphilis and HIV. Laboratory testing was performed, and he was found to have a positive anti-nuclear antibody (ANA) at 1:640 with an elevated sedimentation rate and elevated C-reactive protein. He was referred to pediatric rheumatology for the evaluation of possible autoimmune disease. 

At his initial visit with rheumatology one week later, he complained of joint pain in his knees, ankles, hips, hands, and back. His pain was worse with walking and standing, and he had morning stiffness lasting 1-2 hours and ankle swelling. He endorsed weight loss due to decreased appetite, early satiety, and constipation. He also complained of headaches and had been taking over-the-counter anti-inflammatory medication daily. Family history was notable for SLE in his maternal aunt, maternal grandmother, and great-grandmother. On physical exam, he had swelling and tenderness in multiple proximal interphalangeal joints and the second metacarpophalangeal (MCP) joint in his right hand, swelling in his left knee, and tenderness to motion in his bilateral temporomandibular joints and his left hip. Hand radiographs demonstrated periarticular osteopenia at the MCP joints. Additional laboratory testing showed elevations in anticardiolipin, thyroid peroxidase, and tissue transglutaminase and a positive lupus anticoagulant (Table 1). Antibody testing for double-stranded DNA, extractable nuclear antigens, and beta 2 glycoprotein was negative. His creatinine was mildly elevated, and he had low C4 but normal C3 levels. Urine nucleic acid amplification tests for chlamydia, gonorrhea, and trichomonas were negative. With his clinical examination and laboratory results, a diagnosis of SLE was made, and he was started on hydroxychloroquine 200 mg daily and 60 mg prednisone daily for one week, to taper off over the following three weeks. A baseline ophthalmology examination was normal. 

**Table 1:** Patient laboratory results Laboratory results obtained at different time points during the patient's course of disease. SLE: systemic lupus erythematosus; ANA: anti-nuclear antibody; RPR: rapid plasma reagin; LA: lupus anticoagulant; FTA: fluorescent treponemal antibody

Test name	Reference range	At the time of SLE diagnosis	On admission, 4 months after diagnosis	Outpatient follow-up 1 year later
White blood cells (×10^3^/μL)	4.5-13.0	3.8	4.9	5.2
Hemoglobin (g/dL)	13.5-18.0	13.5	12.2	16.4
Platelets (×10^3^/μL)	142-508	236	255	200
Sedimentation rate (mm/h)	<15	58	47	41
C-reactive protein (mg/dL)	<1.0	1.9	1.9	-
Partial thromboplastin time (s)	24-36	63	50	35
Creatinine (mg/dL)	0.5-1.0 or 1.2	1.12	0.93	1.1
Staclot LA	Negative	Positive	Positive	Negative
ANA screen	Negative	Positive	-	-
ANA titer	<1:40	1:640	-	-
Russell's viper venom test	Negative	Positive	Positive	Positive
Anti-cardiolipin IgG (GPL’U)	0-15	19.6	18.1	<9.4
Anti-cardiolipin IgM (MPL’U)	0-12.5	102.6	124.3	17.5
Anti-thyroid peroxidase (IU/mL)	<35	39	-	-
Anti-tissue transglutaminase IgG (Chm’U)	<20	27	-	-
C3 complement (mg/dL)	86-184	139	130	143
C4 complement (mg/dL)	16-59	11	11	21
HIV 1 multispot	Negative	-	Positive	-
HIV 1 and HIV 2	Negative	-	Positive	-
HIV 2 multispot	Negative	-	Negative	-
HIV 1 quantification (copies/mL)	Not detected	-	323,012	106,739
CD4+ T cells (cells/μL)	511-1,364	-	86	32
RPR	Nonreactive	-	Reactive	Reactive
RPR titer	Nonreactive	-	1:64	1:2
FTA	Nonreactive	-	Reactive	-
*Chlamydia trachomatis* rRNA	Not detected	Not detected	Detected	Not detected
Neisseria gonorrhoeae	Not detected	Not detected	Detected	Not detected

He was seen in the emergency department three weeks after the initial diagnosis with complaints of intermittent shortness of breath, bilateral leg pain, dizziness, and headache. He had just completed his steroid taper the day before presentation. Laboratory results showed a mild increase in creatinine to 1.12 mg/dL, with normal urinalysis, creatinine kinase, liver enzymes, complete blood count, chest radiograph, and electrocardiogram. His symptoms improved with ketorolac and intravenous fluids, and he was discharged home. The day after discharge from the emergency department, he called the rheumatology clinic with complaints of ankle swelling and increased arthralgias, as well as new oral ulcers and a new rash on the palms and soles of the feet that he described as blotchy, dark red spots. He was restarted on prednisone 20 mg daily for one week, and hydroxychloroquine was continued. 

In a follow-up appointment with his rheumatologist three weeks later, his symptoms did improve after starting the prednisone; however, he continued having arthralgia of his fingers, hands, and wrists as well as oral ulcers. He had stopped taking his hydroxychloroquine about one week before this appointment, as he had an episode of tachycardia, dizziness, and headache that he attributed to the medication. At this appointment, he was not noted to have a rash, but did have appreciable swelling in his fingers, knees, and left wrist. Radiographs of the lumbar spine and hips were normal. He was started on subcutaneous methotrexate weekly; however, he became ill with SARS-CoV-2 infection and did not start taking this medication for another 2-3 weeks. 

At his rheumatology appointment the following month, his symptoms had worsened despite having started methotrexate. He had developed superficial tongue ulcers, and the rash on the palms and soles had returned. He had continued general arthralgias with difficulty dressing himself and jaw pain with chewing. His folic acid dose was increased from 1 mg to 2 mg, and prednisone 20 mg daily was restarted. In the following weeks, he called his rheumatologist requesting an appointment for worsening arthritis. He was seen urgently for symptoms of sinus pressure, sore throat, constipation, nausea, jaw pain, and hair loss. On exam, he was noted to have prominent oral ulcers on the hard palate with white plaques on the buccal mucosa. He had bilateral knee effusions, hair thinning over his temporal region, and erythema of the palms and soles with a faint rash over the bridge of the nose. Given the severity of his symptoms, he was admitted to the hospital with plans for intravenous pulse methylprednisolone. 

Upon admission, additional history was obtained, and he reported sexual activity with inconsistent condom use and a new male partner who was taking pre-exposure prophylaxis for HIV. Laboratory testing was performed and detected syphilis via rapid plasma reagin (RPR) and HIV 1 in the serum and gonorrhea and chlamydia in the urine. He was treated for syphilis with a dose of intramuscular penicillin G and plans for two further doses. His oral ulcers were felt to be more likely due to oral candidiasis based on his low CD4 count, and this was treated with micafungin. His gonorrhea infection was treated with ceftriaxone, and his chlamydia with doxycycline. When confirmatory HIV testing was positive, antiretroviral therapy with bictegravir, emtricitabine, and tenofovir alafenamide was planned, and trimethoprim-sulfamethoxazole prophylaxis was prescribed given his low CD4 count (86 cells/µL). Due to these infections, methotrexate and folic acid were discontinued. After discussion between his rheumatologist and HIV physician, his SLE therapy was held until his syphilis therapy was completed, and a prednisone taper was initiated. 

Over the subsequent months, the patient was lost to follow-up. He took only one month of antiretroviral medication, did not start *Pneumocystis* prophylaxis, and did not receive his second or third doses of penicillin G. About one year after his admission, he presented to an urgent care with a headache and joint pains in his bilateral hips and ankles that were worse in the morning, which he attributed secondary to SLE. He also endorsed shortness of breath with activity and a hyperpigmented rash on his shoulder and genital area. Tenderness, but no joint swelling, was noted on his exam at this time, and he left without the recommended medical workup.

The patient attended outpatient follow-up in the HIV clinic a few weeks later, where labs were significant for continued elevation of HIV viral load and decreased CD4 counts, but improving anticardiolipin IgM titer (Table 1). His C3, C4, double-stranded DNA, and extractible nuclear antigen testing remained normal. He was restarted on bictegravir, emtricitabine, and tenofovir alafenamide, as well as trimethoprim-sulfamethoxazole. Further doses of penicillin G were deferred due to appropriately low RPR titers, and SLE treatment was continued to be held until HIV infection was controlled. At a follow-up visit after completing one month of antiretroviral treatment, his HIV viral load was nearly undetectable, and his rash was resolving; however, he continued to have diffuse joint pain primarily in his hands and wrists. He was instructed to continue follow-up in the HIV clinic, which he has done, but he has been lost to rheumatology follow-up. 

## Discussion

**Figure 1:**
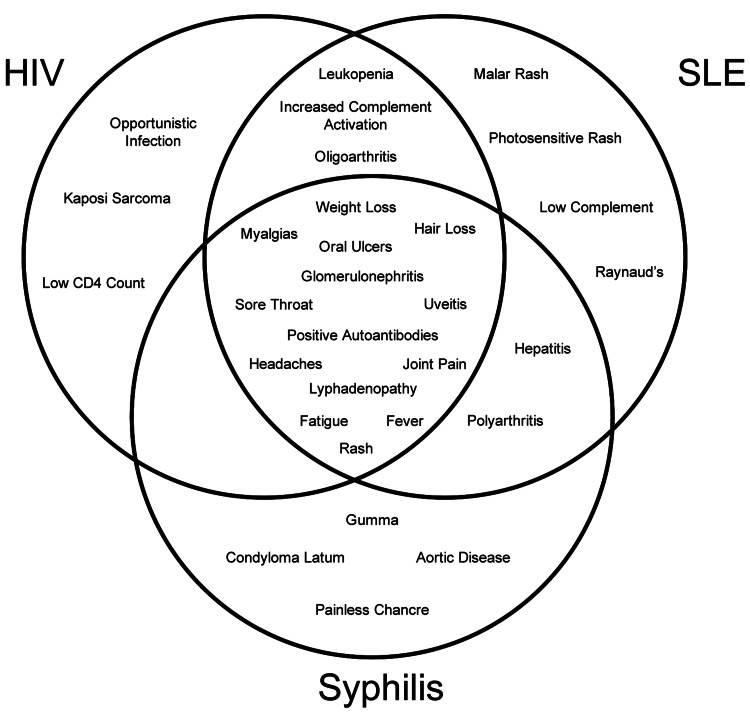
Venn diagram illustrating the overlap in features between HIV, SLE, and syphilis This Venn diagram helps illustrate the overlap in features between the three entities, as well as some key distinguishing features for each. HIV: human immunodeficiency virus; SLE: systemic lupus erythematosus Image Credit: Alison Brittain

This case highlights the degree of overlap in clinical symptoms between SLE and certain STIs, especially HIV and secondary syphilis. This overlap can include similar laboratory findings such as false-positive autoantibody testing, leukopenia, or elevations in liver function testing, as well as clinical symptoms including rashes, joint pain and swelling, lymphadenopathy, and constitutional symptoms (Figure 1). Reactive arthritis can also present in patients with HIV or in those with bacterial gastrointestinal and genitourinary infections including *Chlamydia trachomatis*. These similarities make testing for STIs an essential part of the diagnostic workup of rheumatologic disease in adolescents. 

Much of the diagnostic confusion between SLE and HIV lies in the ability of HIV to cause arthropathy and skin lesions typical of autoimmune disease. The arthropathy of HIV is most commonly a symmetric oligoarticular arthritis of the lower extremities and is usually more acute in onset than SLE [7]. Several studies have also shown an increased incidence of psoriasis and psoriatic arthritis in patients with HIV infection [8-11]. Unusually severe and atypical presentations of psoriatic arthritis have been attributed to HIV infection, particularly as CD4 counts drop below 500 cells/μL.

HIV can also mimic SLE in its ability to cause positive autoantibody tests, including the formation of anti-double-stranded DNA, anti-RNA, anti-cardiolipin, anti-phospholipid, anti-tissue transglutaminase, and anti-thyroid peroxidase antibodies [4]. Both SLE and HIV are associated with the activation of the complement cascade; however, unlike SLE, HIV infection is not known to be associated with decreased C3 or C4, which may help distinguish between the two entities [12]. Also, unlike SLE, HIV is not commonly associated with a malar rash, which may aid in differentiating the two. 

While concurrent diagnosis of HIV and SLE is rare, about ~60 cases have been documented in the literature [13-16]. Many of these cases were thought to be drug-induced lupus caused by antiretroviral therapy or immune reconstitution syndrome leading to immune system overactivation and subsequent SLE diagnosis. Similar to this case, there have also been reports of HIV infection being misdiagnosed as SLE due to symptom overlap [17]. When making the diagnosis of HIV infection, it is also prudent to remember that seroconversion occurs 3-12 weeks after infection and infection may not be detected if testing is done too soon following exposure. CD4 counts also do not typically fall until years after primary infection, which suggests that our patient either was infected for several years and did not have HIV testing or had false-negative testing or possibly the use of immunosuppressive therapies influenced his CD4 cell count. 

In the case of our patient, in the absence of the co-occurring STI, he would have met EULAR/ACR criteria for SLE, which underscores the importance of thoroughly ruling out these infections before diagnosis. He had some of the more distinguishing symptoms of SLE, including several positive autoantibodies (anti-cardiolipin, anti-thyroid peroxidase, anti-tissue transglutaminase) and low complement, though his low complement resolved without adequate treatment for HIV or SLE. At follow-up one year later, his anti-cardiolipin IgG testing had returned to normal, and his anti-cardiolipin IgM titer was greatly reduced, suggesting that these two autoantibodies were likely reactive to infection. His other autoantibody tests have not been repeated yet. Our patient also had a positive family history for SLE, further pointing physicians to a rheumatologic diagnosis. It remains to be seen what symptoms of SLE, if any, will remain after adequate treatment for HIV infection. 

To complicate this case further, our patient was diagnosed with three other STIs, with syphilis having the greatest symptom overlap with SLE. A few previous case reports have documented the clinical challenge of distinguishing symptoms of syphilis, particularly secondary syphilis, from SLE [18,19]. Like SLE, *Treponema pallidum* infection can cause positive autoantibody testing (particularly of anti-phospholipid antibodies) and manifest with a broad range of non-specific symptoms [20]. The overlap between the presentation of SLE and syphilis is strongest during the secondary stage of syphilis. This stage generally starts about six weeks after primary infection and is characterized by low-grade fever, weight loss, lymphadenopathy, rash, myalgias, sore throat, hair loss, fatigue, and, in rare cases, polyarthritis, hepatitis, and glomerulonephritis. Each of these symptoms can also be seen in SLE, and many of these symptoms were exhibited by our patient. Typically, secondary syphilis presents with a palmoplantar macular or papular rash, as seen in our patient. Our patient's lack of improvement with methotrexate and emergence of oral lesions and rash after starting therapy were also red flags suggesting a different diagnosis rather than SLE. However, after adequate treatment for syphilis, gonorrhea, and chlamydia, our patient still had joint pains and rash, suggesting either HIV or SLE was responsible for these symptoms. 

## Conclusions

The case described above highlights the need to keep a broad differential when diagnosing SLE and to perform the appropriate testing to rule out other entities before final diagnosis or escalation of immunosuppressive therapy. SLE, HIV, and syphilis all share similar features that can cause diagnostic error and treatment delay if not appropriately ruled out before making a single final diagnosis or if these entities present concomitantly and not all are promptly identified. Other etiologies should always be considered when SLE flares are not responding to conventional therapy or when symptoms of autoimmune disease persist despite adequate treatment of infection. In addition, STI testing should be performed in all at-risk patients based on guidance from the Centers for Disease Control and Prevention (CDC) or other relevant society guidelines particularly before starting immunosuppressive therapies. 
